# Cardiovascular benefits and safety profile of acarbose therapy in prediabetes and established type 2 diabetes

**DOI:** 10.1186/1475-2840-6-20

**Published:** 2007-08-15

**Authors:** Markolf Hanefeld

**Affiliations:** 1 Zentrum für Klinische Studien, GWT, Technische Universität Dresden, Dresden, Germany

## Abstract

Dysglycaemic disease is one of the most important health issues facing the world in the 21^st ^century. Patients with type 2 diabetes and individuals with prediabetes are at risk of developing macrovascular and microvascular complications. Long-term management strategies are therefore required that are effective at controlling dysglycaemia, well tolerated and, ideally, offer additional cardiovascular disease (CVD) risk-reduction benefits. The efficacy, safety and tolerability of the α-glucosidase inhibitor acarbose have been well-established in a wide range of patient populations in both clinical and community trials. In addition, acarbose has been shown to reduce cardiovascular complications in type 2 diabetes and prevent hypertension and CVD in individuals with impaired glucose tolerance (IGT). Acarbose has a very good safety profile and, owing to its straightforward, non-systemic mode of action, avoids most adverse events. The most common side-effects of acarbose are mild-to-moderate gastrointestinal complaints that subside as treatment continues. They can be minimised through the use of an appropriate stepwise dosing regimen and careful choice of diet. Acarbose is therefore a valuable option for the management of type 2 diabetes and, as the only oral antidiabetes agent approved for the treatment of prediabetes, can help to improve clinical management across the dysglycaemic disease continuum.

## Background

Cardiovascular disease (CVD) is the leading cause of mortality associated with dysglycaemia. Type 2 diabetes accounts for almost one in ten of all deaths around the world each year, and up to 80% of these deaths are CVD-related [1,2]. Moreover, there is now substantial evidence to show that the prediabetic state of impaired glucose tolerance (IGT) is itself a significant CVD risk factor [3,4].

Several management strategies have been proposed for the early stages of dysglycaemia, with the aim of preventing the development of type 2 diabetes and associated complications, such as CVD. A key strategy is "lifestyle modification", involving changes in diet and exercise, which was shown in both the US Diabetes Prevention Program (DPP) and the Finnish Diabetes Prevention Study (DPS) to reduce the incidence of type 2 diabetes by 58% [5,6]. However, although lifestyle modification is a vital part of dysglycaemia management, it is often insufficient to maintain long-term glycaemic control. In such cases, pharmacological intervention will be required; such treatment should place the minimum additional strain on a patient's metabolic, endocrine and vascular systems. Consequently, several antidiabetes and other medications have been studied to determine their potential benefits in the prevention of type 2 diabetes.

Results from trials of acarbose, metformin and rosiglitazone in prediabetic populations are summarised in Table 1. The Study to Prevent Non-Insulin Dependent Diabetes Mellitus (STOP-NIDDM) found that treatment with acarbose reduces the incidence of type 2 diabetes by 36% [7]. In the DPP, treatment with metformin reduced the incidence of diabetes by 31%, although this effect was less marked in older patients, perhaps owing to age-related differences in insulin secretion [5,8]. The DPP also found a significant reduction in the incidence of diabetes with troglitazone, another insulin sensitiser; however, troglitazone was discontinued before the end of the study owing to concerns regarding liver toxicity [9]. More recently, the structurally related drug rosiglitazone was found to reduce the risk of type 2 diabetes and increase reversion to normal glucose tolerance when administered in addition to lifestyle modification [10]. Although no liver toxicity was observed in this study, patients receiving rosiglitazone did have a significantly increased risk of chronic heart failure (p = 0.01) [11].

**Table 1:** Use of oral antidiabetes drugs to treat individuals with prediabetes: effect on cardiovascular and diabetes outcomes.

Study	DREAM [94] (Rosiglitazone)	STOP-NIDDM [7,20] (Acarbose)	DPP [5,48]
Patient population	IGT/IFG n = 5269	IGT n = 1368	IGT n = 2155
Effect of intervention on diabetes incidence	-60% (p < 0.0001)	-36.4% (p = 0.003)	-31% (p < 0.01)
Effect of intervention on new hypertension incidence	n/a	-34% (p = 0.006)	+25% (not significant)
Effect of intervention on rate of predefined CV events	+37% (p = 0.08)	-49% (p = 0.03)	n/a

DREAM: Diabetes Reduction Assessment with Ramipril and Rosiglitazone Medication. STOP-NIDDM: Study to Prevent Non-Insulin Dependent Diabetes Mellitus. DPP: Diabetes Prevention Program

Some cardiovascular drugs have also shown potential antidiabetes effects. Bezafibrate, which lowers triglyceride levels and raises high-density lipoprotein (HDL)-cholesterol levels, has been shown to delay the onset of type 2 diabetes in obsese patients and patients with IGT [12,13]. A recent meta-analysis concluded that the use of renin-angiotensin system antagonists (angiotensin-converting enzyme [ACE] inhibitors or angiotensin receptor blockers [ARBs]) may contribute to the prevention of type 2 diabetes [14]. Equally, a number of recent trials have focussed on the potential of oral antidiabetes drugs to reduce cardiovascular risk, although in most cases further research is needed [15].

Increasing evidence supports the benefits of early treatment with α-glucosidase inhibitors such as acarbose. These agents directly target postprandial hyperglycaemia, which has been identified as an important cardiovascular risk factor in its own right [16,17]. As a result of their non-systemic mode of action – they are active in the gut and not absorbed into the body – α-glucosidase inhibitors are considered to be one of the safest and best-tolerated classes of antidiabetes agents available [18].

The efficacy of acarbose has been confirmed in more than 350 studies involving more than 30,000 patients [19]. Acarbose has been shown to be as efficacious as other commonly used antidiabetes agents, and to have an excellent safety profile with minimal drug-drug interactions. It has been proven to reduce the incidence of type 2 diabetes and hypertension in people with IGT [7,20]; in addition, studies in both prediabetes and type 2 diabetes populations have demonstrated that the use of acarbose is associated with reductions in cardiovascular events [20,21] and with beneficial effects on a broad spectrum of CV risk factors. Acarbose is currently the only oral antidiabetes agent approved for the treatment of both prediabetes and type 2 diabetes.

### Direct targeting of postprandial hyperglycaemia

Postprandial hyperglycaemia is known to contribute to the development of endothelial dysfunction [22,23], and to increase the risk of cardiovascular and all-cause mortality, even before diabetes is diagnosed [17,24,25]. Hyperglycaemia is also one of the metabolic abnormalities that defines metabolic syndrome [25,26]. Patients with prediabetes and type 2 diabetes are therefore more likely than the general population to develop dyslipidaemia and hypertension. The International Diabetes Federation (IDF) has estimated that approximately 500 million people worldwide suffer from some degree of dysglycaemia [27,28]. To meet the healthcare challenges posed by this epidemic, diabetologists and primary care physicians need to be aware of the most appropriate treatment options for all stages of the disease continuum.

**Figure 1:**
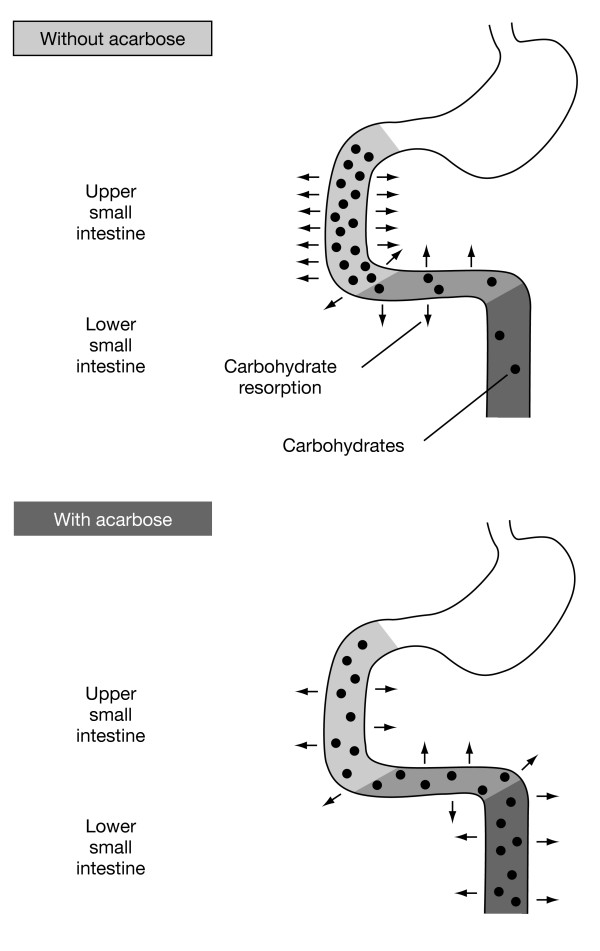
Acarbose delays the absorption of carbohydrates from the gut.

Acarbose binds with high affinity and specificity to α-glucosidases found in the brush border of the small intestine. Under normal circumstances, these enzymes are responsible for the hydrolysis of complex carbohydrates (starch and other oligosaccharides) to absorbable simple sugars (monosaccharides, most importantly glucose). Alpha-glucosidases are found along the length of the intestine, but in Western populations current dietary habits typically mean that in most people they are only active in the upper small intestine. The effect of acarbose is to delay the digestion of oligosaccharides in the small intestine so that the release and absorption of glucose takes place over a longer time across the length of the small intestine (Figure 1) [29]. The result is that acarbose therapy directly reduces postprandial hyperglycaemia [30]. A Cochrane review of 30 acarbose monotherapy trials, which included more than 5,000 patients with type 2 diabetes, demonstrated that acarbose treatment reduces 1-hour postload plasma glucose levels by 2.3 mmol/L with a clear dose-response relationship, as well as fasting plasma glucose (FPG) levels and HbA_1c _levels – a measure of long-term glycaemic control [31].

In 24-week face-to-face comparison trials, similar reductions in the level of glycated haemoglobin (HbA_1c_) were seen for acarbose and metformin (1.1% and 1.0%, respectively) [32], and for acarbose and glibenclamide (1.1% and 0.9%, respectively) [33]. The Cochrane review  also found that acarbose is at least as effective at controlling  postprandial hyperglycaemia as commonly used oral antidiabetics,  short-acting insulin secretagogues and the long-acting sulphonylurea  glibenclamid  [31]. The natural progression of diabetes involves increasing insulin resistance and loss of pancreatic β-cell function, and antidiabetes therapies that trigger insulin secretion therefore fail over time. The mechanism of action of acarbose, however, limits the burden on β-cells, and studies have shown that the effectiveness of acarbose is maintained in long-term treatment, with no secondary failure. A 5-year surveillance study of patients with diabetes found that acarbose reduced 2-hour postprandial blood glucose (2 hPG) levels by 3.4 mmol/L and HbA_1c _levels by 1.8% from baseline [18,34]. By contrast, the effectiveness of some other oral antidiabetic drugs, such as glibenclamide, is dependent on adequate β-cell function, and is therefore likely to decline as diabetes progresses, as shown in the UKPDS [35] and other studies [18,36].

### Preventing cardiovascular disease with acarbose

The link between dysglycemia – and postprandial hyperglycaemia in particular – and cardiovascular risk is well established [17,37-39]. Type 2 diabetes, for instance, is a reliable independent predictor of mortality after a myocardial infarction (MI), [40] and blood glucose levels have been shown to be a continuous risk factor for CVD mortality even below the diabetic threshold [41,42].

The cardiovascular benefits of acarbose in individuals with IGT were shown by STOP-NIDDM. The study found that the reduction in postprandial hyperglycaemia with acarbose therapy was not only associated with a 36% reduction in newly diagnosed diabetes, but also with a 34% relative risk reduction in the incidence of new cases of hypertension (*P *= 0.006) and a 49% relative risk reduction in the development of cardiovascular events (*P *= 0.03) over a mean follow-up of 3.3 years (Table 1) [20]. Moreover, the risk of MI specifically was reduced by 91% (*P *= 0.02), a result that remained significant even when an additional eight silent MIs were identified and included in the analysis (total of two clinically overt and silent MIs in the acarbose group, compared with 19 in the placebo group; *P *= 0.001)[43].

**Figure 2:**
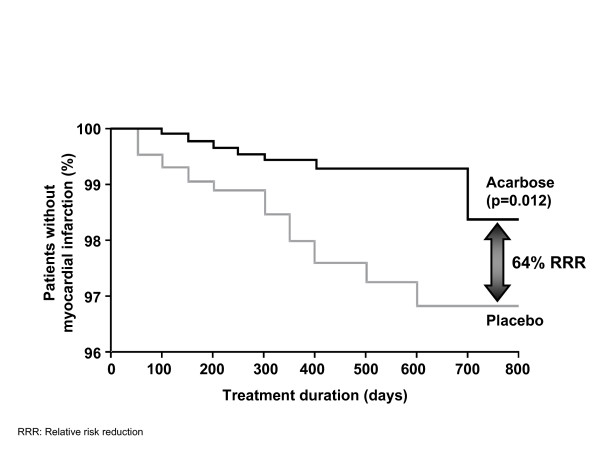
Acarbose reduces the risk of myocardial infarction in patients with type 2 diabetes. [21]

Other oral antidiabetes agents used for the prevention of diabetes in patients with IGT have not been shown to produce similar reductions in CVD risk. In the recent DREAM (Diabetes REduction AssessMent with ramipril and rosiglitazone) study, the effect of treatment with the thiazolidinedione rosiglitazone on the incidence of cardiovascular events in prediabetic individuals was assessed as a secondary endpoint. The composite cardiovascular endpoint was not significantly different between the two groups (hazard ratio [HR], 1.37; 95% confidence interval [CI], 0.97–1.94; *P *= 0.08)[44]. However, despite the fact that the study was carried out in a population at low risk for cardiovascular events, significantly more participants developed congestive heart failure in the rosiglitazone group than in the placebo group (14 vs 2 participants, respectively; HR, 7.03; 95% CI, 1.60–30.9; *P *= 0.01)[45]. The MEta-analysis of Risk Improvement under Acarbose (MeRIA), which analysed the results of seven long-term trials carried out in patients with type 2 diabetes, confirmed that treatment with acarbose has broader benefits for the management of vascular disease and damage [21]. The primary endpoint of the MeRIA analysis was the time to develop a cardiovascular event. Treatment with acarbose reduced the risk of any cardiovascular event by 35% (*P *= 0.0061), and the risk of MI specifically by 64% (*P *= 0.0120; Figure 2) [21]. Trends towards risk reduction were observed for all other selected cardiovascular events, including angina, heart failure and stroke.

The effects of a thiazolidinedione, pioglitazone, on the secondary prevention of cardiovascular events in patients with type 2 diabetes was assessed in PROactive (PROspective pioglitAzone Clinical Trial In macrovascular eVEnts); this study also failed to show a significant reduction in the primary CV endpoint with treatment [46]. In the UKPDS, metformin was shown to reduce the incidence of myocardial infarction (*P *= 0.010) and all-cause mortality (*P *= 0.011) in obese type 2 diabetes patients [47]. However, in the Diabetes Prevention Programme, which randomised 3,234 individuals with IGT to intensive lifestyle modification, metformin or placebo, there was no difference in the incidence of cardiovascular events between the treatment groups over 3 years [48].

### Improvements in cardiovascular risk factors with acarbose

**Figure 3:**
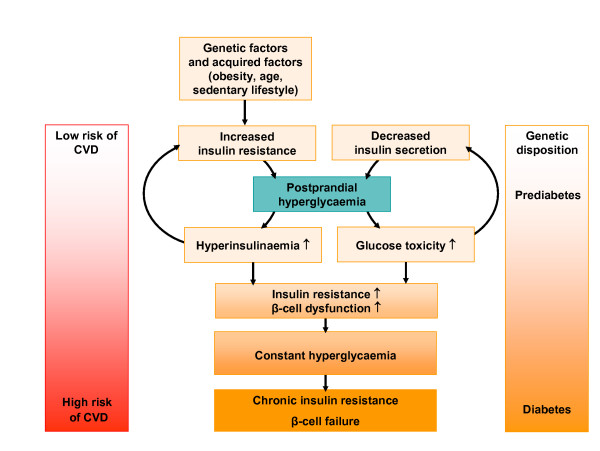
The role of postprandial hyperglycaemia in the development of type 2 diabetes and CVD.

The rapid rise in postprandial blood glucose levels associated with IGT damages the endothelium of the arterial wall and initiates a cascade of pro-atherogenic events (Figure 3) [23,49]. Research is ongoing to determine the mechanisms by which acarbose provides cardiovascular benefits, but it seems clear that the benefits can be attributed, at least in part, to the fact that acarbose directly targets postprandial hyperglycaemia, which limits endothelial damage and reduces the risk of macrovascular complications. For example, a substudy of STOP-NIDDM found that acarbose slows the progression of intima-media thickening (IMT) in individuals with prediabetes; compared with placebo, acarbose treatment reduced the annual increase in carotid IMT by approximately 50% (*P *= 0.027)[22]. This result is supported by the results of a subsequent study in which acarbose treatment was shown to preserve endothelial vasodilation compared with placebo [23].

Patients with type 2 diabetes are at high risk for the other components of metabolic syndrome, including dyslipidaemia and hypertension, which have been shown to lead to increased morbidity and mortality [26,50]. It has been suggested that management of metabolic syndrome should focus on the prevention of diabetes and CVD [51], and treatment with acarbose has been shown to have benefits for several metabolic syndrome components.

For example, in contrast to other oral antidiabetes agents, acarbose is not associated with weight gain. The use of sulphonylureas, glinides or thiazolidinediones to manage type 2 diabetes is associated with a typical weight gain of 2–5 kg [52], but a randomised controlled comparison of acarbose and glibenclamide as monotherapy in 96 patients with diabetes found no weight changes associated with acarbose therapy [32]. Moreover, a 22-week trial found that acarbose therapy caused a significant reduction in weight compared with placebo (*P *< 0.01)[53], a result confirmed by the MeRIA analysis [21]. Nor does acarbose therapy lead to weight gain in patients with prediabetes: in STOP-NIDDM, after 3.2 years, individuals receiving acarbose had lost a mean of 1.2 kg, compared with individuals receiving placebo [7]. These benefits cannot be explained by changes in dietary habits [54,55].

**Figure 4:**
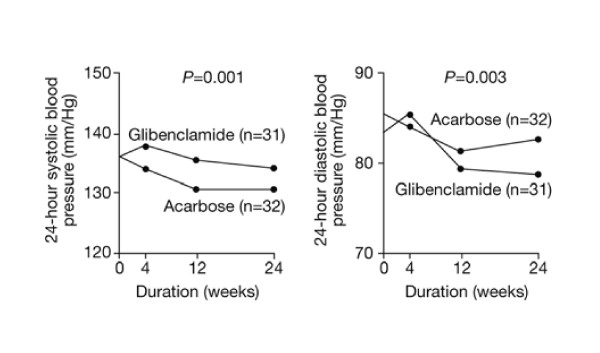
Mean systolic and diastolic blood pressure in patients with hypertension and type 2 diabetes treated with acarbose or glibenclamide for 6 months. Adapted with permission from Rosenthal & Mausersberger. [57]

Between 20% and 60% of patients with type 2 diabetes will develop hypertension [56]. A double-blind, randomised, placebo-controlled study in 44 patients with type 2 diabetes found that achievement of good glycaemic control with acarbose was accompanied by significant reductions in diurnal systolic, diastolic and mean blood-pressure values (*P *< 0.05)[53]. Similarly, a randomised 6-month study in obese patients with diabetes found that acarbose treatment reduced the mean 24-hour systolic blood pressure by a mean of 5.2 mmHg, compared with only 1.6 mmHg with glibenclamide (*P *= 0.0001, Figure 4) [57]. Again, these results were confirmed by the MeRIA analysis, which found that acarbose treatment significantly reduces systolic blood pressure (*P *= 0.024)[21,58].

Reductions in postprandial hyperglycaemia in patients with type 2 diabetes as a result of treatment with acarbose have also been shown to reduce the activity (*P *= 0.045) and nuclear localisation (*P *= 0.02) of the proinflammatory transcription factor NFκB [59], suggesting a mechanism by which the anti-inflammatory effects of acarbose may be mediated. This mechanism would be consistent with reductions in the levels of coagulation factors seen with acarbose treatment. For example, acarbose has been shown to reduce the level of fibrinogen in patients with type 2 diabetes (*P *= 0.013 vs placebo)[60], and to reduce serum C-reactive protein levels in individuals with IGT (*P *< 0.01 vs placebo)[61]. However, unlike acarbose, most treatments for type 2 diabetes, such as metformin and sulphonylureas, have a greater effect on FPG levels than on postprandial hyperglycaemia [62], and may not lead to cardiovascular benefits by these mechanisms. Furthermore, an analysis of 24-hour insulin and proinsulin profiles in type 2 diabetes patients treated with either acarbose or glibenclamide found that acarbose significantly reduced diurnal levels of proinsulin (*P *< 0.05)[63]. Improvement of insulin sensitivity by acarbose has been shown with hyperglycaemic clamp measurements in elderly patients with type 2 diabetes [64].

### Side-effects and tolerability of acarbose

Numerous clinical and surveillance studies have confirmed that, in contrast to other antidiabetes medications, in particular thiazolidinediones, acarbose treatment is associated with very few serious side-effects. The 28-week Precose Resolution of Optimal Titration to Enhance Current Therapies (PROTECT) trial, which followed more than 6,000 patients with type 2 diabetes in a real-world setting, concluded that acarbose has an excellent safety profile irrespective of patients' age, weight, ethnicity, the time since their diagnosis and the presence or absence of concomitant therapy [65].

The most common side-effects seen with acarbose are mild-to-moderate gastrointestinal (GI) side-effects, including flatulence, diarrhoea and abdominal distension. In phase 3 trials in the USA, 56–76% of patients receiving acarbose reported side-effects, of which almost all were GI-related. However, side-effects were also reported by 32–37% of patients receiving placebo [18,66]. In addition, a 24-week study of 495 patients with diabetes from seven European countries found that, of patients who received acarbose and reported flatulence during the first 2 weeks of therapy, only about half reported flatulence during the last 4 weeks [18,67]. Most GI sideeffects, therefore, become less common as therapy continues [18,66]. A 12-week postmarketing surveillance study of 27,803 patients with diabetes found that 13.7% reported flatulence and 2.2% reported diarrhoea; 83% of patients reported no adverse reactions [18,68]. Furthermore, a 5-year surveillance study of 1,954 patients with diabetes found that GI side-effects associated with acarbose were reported by only 3.9% of patients (Table 2)[18,69].

**Table 2:** Safety profile of acarbose, 5-year surveillance study (n = 1,954) [34].

Side-effect	n	Proportion of total population (%)
**Gastrointestinal**	**76**	**3.9**
Flatulence	68	3.5
Diarrhoea	13	0.7
Loose stool	1	<0.1
Abdominal	4	0.2
Nausea	3	0.2
Constipation	1	<0.1
**Metabolic**	**10**	**0.5**
**Body as a whole**	**5**	**0.3**

The GI side-effect profile of acarbose is linked to the drug's mechanism of action. In many individuals, α-glucosidases are most active in the upper small intestine. As a result, in the early stages of therapy, some undigested carbohydrate reaches the colon. There, it undergoes fermentation by colonic bacteria, resulting in the formation of fatty acids, which are absorbed by the body, and gaseous byproducts, which cause GI side-effects. As acarbose treatment continues, there is a compensatory increase in enzyme activity in the lower small intestine [18,69]. Furthermore, exposure of the lower small intestine to undigested carbohydrate leads to an increased quantity and duration of glucagon-like peptide-1 (GLP-1) release [70]. These adaptations explain the reduction in GI side-effects over time.

Patient compliance with pharmacological therapy is affected by both the efficacy and tolerability of the drugs taken. A double-blind, placebo-controlled substudy of acarbose in 1,946 patients was initiated 4 years before the end of the UKPDS; it found that most discontinuations from acarbose therapy occurred during the first year of treatment [71], and in the 5-year open-label surveillance study, two-thirds of side-effects were reported in the first 3 months [34]. In STOP-NIDDM, which recruited 1,429 individuals with IGT, 48% of those who discontinued early did so in the first year, and the most common single cause of discontinuation was GI side-effects [7]. Trial results indicate that initial intolerance owing to GI side-effects is transient, and that once tolerance is achieved, compliance is easier to maintain. In addition, owing to its mechanism of action, the effectiveness of acarbose does not decrease over time.

**Figure 5:**
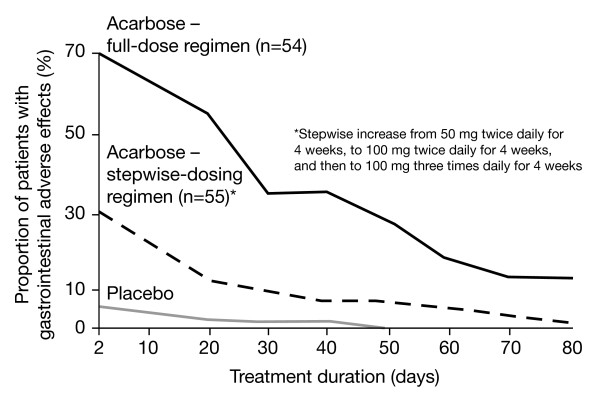
Numbers of patients with intestinal side-effects receiving placebo (n = 55), 100 mg acarbose (n = 54), and 100 mg acarbose with stepwise dosing (n = 55) during 12-week treatment. Adapted with permission from May. [72]

The side-effect profiles of two different acarbose dosing regimens, compared with placebo, were determined in a multicentre trial of 164 outpatients with type 2 diabetes [72]. A stepwise-increasing regimen, in which acarbose therapy was initiated at 50 mg twice daily and progressed to 100 mg three-times daily, was found to be as effective at reducing postprandial glucose levels as a flat-dosing regimen, in which patients received 100 mg three-times daily from the outset. Both regimens significantly reduced 2 hPG and mean HbA_1c _levels (both *P *< 0.0001 vs placebo). However, the stepwise-dosing regimen was associated with significantly fewer GI side-effects (Figure 5, *P *< 0.05) that were significantly less persistent than the flat-dosing regimen over the course of treatment. The mean duration of GI side-effects for patients in the stepwise-dosing group was 29 days, compared with 21 days in the placebo group and 42 days in the flat-dosing group (*P *< 0.05)[72]. A 'start low, go slow' stepwise-dosing regimen is, therefore, recommended to minimise side-effects [73,74]. To minimise the disruptive effects of any GI side-effects, depending on regional dietary habits, some physicians recommend that when initiating acarbose it may be more effective to take the initial once-daily dose with the evening meal, rather than with breakfast [75].

In STOP-NIDDM, administration of acarbose was initiated at 50 mg/day and titrated by 50 mg/day every 2 weeks to a maximum of 100 mg three-times daily (or the maximum tolerated dose)[76]. The same regimen was used in the UKPDS [71] and a similar regimen was used in a Chinese study [77], whereas in the PROTECT trial acarbose was titrated from 25 mg three-times daily to 50 mg three-times daily, and finally to 100 mg three-times daily based on efficacy and tolerability [65]. In all these trials, acarbose was shown to effectively reduce hyperglycaemia, and in STOP-NIDDM, acarbose also significantly increased reversion of IGT to normal glucose tolerance (*P *< 0.0001)[7]. Furthermore, these trials demonstrated that the full maximum dose (300 mg three-times daily) is not required in all patients. The mean daily dose in STOP-NIDDM was 194 mg [7], compared with 201 mg in the 5-year surveillance study [34]. The low prevalence of side-effects in the surveillance study may be explained by the physician tailoring the dose of acarbose to the individual patient in a community setting [34].

Other factors can also impact on the effectiveness of acarbose treatment, and therefore potentially affect compliance. Enzyme activity in the gut varies among individuals and among populations, and can lead to variations in the initial response to acarbose treatment [75]. In addition, because the effects of acarbose are the result of competitive inhibition, acarbose must be present at the active site of its target enzymes at the same time as the ingested carbohydrates. Maximal effectiveness is therefore achieved if acarbose is given within 15 minutes of the start of a meal, and it is recommended that acarbose is taken with the first bite of a meal [78].

The carbohydrate composition of a meal can also affect the efficacy and tolerability of acarbose therapy. A diet high in complex carbohydrates and low in simple sugars is recommended for most patients with diabetes, but is particularly important for patients receiving acarbose [79]. A very high intake of simple sugars may aggravate GI side-effects, because they may be present in too high a concentration to be absorbed in the upper small intestine. Therefore, if side-effects are reported, dietary management will help to overcome them. Physicians and nurses should explain that GI symptoms are part of the mode of action of acarbose, and usually disappear with continuous treatment.

### Safety profile of acarbose

The mechanism of action of acarbose, and its unique, non-systemic pharmacology, avoids several common adverse events associated with other antidiabetes therapies, and consequently, acarbose has few contraindications [18,66]. For example, hypoglycaemia, which is caused by an excess of insulin, is a limiting factor in near-to-normal control of hyperglycaemia [80,81], and is a significant cause of morbidity and mortality [82]. Due to its mode of action, acarbose does not stimulate insulin secretion, and therefore monotherapy is not expected to cause hypoglycaemia [81]. The UKPDS found that there was no difference between acarbose and placebo for the risk of major or minor hypoglycaemic episodes [71]. By contrast, the use of insulin secretagogues (sulphonylureas and glinides) is associated with an increased risk of hypoglycaemia [52]. In the UKPDS, the incidence of any hypoglycaemic episode with sulphonylurea therapy was 17.7% [83]; in general, the incidence of severe hypoglycaemia with this therapy class is estimated to be 1–2% per year [52].

Acarbose is poorly absorbed into the bloodstream, and has a low systemic availability of less than 2% [84]. As a result, the risk of any toxic reaction is very low and, to date, no interactions have been reported between acarbose and β-blockers, sulphonylureas, angiotensin-converting enzyme (ACE) inhibitors or warfarin therapy [18]. Some other antidiabetes agents are contraindicated with other treatments; for example, concomitant use of repaglinide with gemfibrozil, a lipid-lowering agent that acts by inhibiting cytochromes, can cause prolonged hypoglycaemia [52].

Acarbose has been associated with rare cases of hepatotoxicity [85] and increased liver enzyme levels [66], but these have not been confirmed in major trials. A few cases of fulminant hepatitis with fatal outcome have been reported [86,87]; but a causal relationship with acarbose treatment has not been clearly established [85]. A trial of 20 patients with diabetes and either chronic hepatitis or liver cirrhosis found that acarbose significantly decreased the level of HbA_1c_, from 7.2% at entry to 6.3% (*P *< 0.05) after 8 weeks [88]. More recently, a larger trial of 107 cirrhotic patients with diabetes found that, over 8 weeks, acarbose reduced postprandial glucose levels by about 50%, with no changes in liver function [89]. By contrast, sulphonylureas, metformin and some thiazolidinediones are contraindicated in patients with moderate-to-severe liver dysfunction [52,65].

Diabetes and many diabetic complications are particularly common in elderly individuals [90]. Acarbose has been safely used in elderly patients. A randomised controlled trial of 192 patients with type 2 diabetes aged ≥ 65 years found that acarbose monotherapy controlled blood-glucose levels, with no cases of hypoglycaemia reported and no clinically relevant changes in vital signs during the study [91]. A large postmarketing surveillance study confirmed that acarbose is effective at controlling blood-glucose levels in elderly patients, and also found that the incidence of adverse events with acarbose was low and independent of age [68]. Clinical observations also show that elderly patients receiving acarbose report fewer problems with constipation.

Diabetes accounts for 44% of new cases of end-stage renal disease, making it a leading cause of nephropathy [92]. However, there are few data available regarding the use of acarbose in patients with renal disease. Current recommendations therefore state that acarbose should not be given to patients with severe renal impairment. A number of other therapies are contraindicated in this patient population; for example, the use of metformin in patients with renal impairment is associated with an increased risk of lactic acidosis, which can be fatal [36].

The long-term safety of acarbose has been demonstrated in placebo-controlled trials and in postmarketing surveillance studies. In the randomised, double-blind UKPDS, patients visited hospital-based clinics at 4-month intervals. Acarbose, in combination with other antidiabetes therapies, maintained a good safety profile over 3 years [71]. A 2-year, more-closely monitored trial in 74 patients with diabetes reported a similar level of adverse events as the UKPDS, and no serious adverse events were considered to be related to acarbose [93]. Safety in an IGT population was demonstrated by STOP-NIDDM, over a mean follow-up of 3.2 years [7]. By extrapolation – and in contrast to treatment with metformin or thiazolidine – no laboratory safety monitoring is required when treatment with acarbose is initiated.

## Conclusion

Numerous trials have demonstrated that acarbose is a safe and effective oral antidiabetes agent in patients with diabetes and individuals with IGT. These findings have been confirmed in a wide range of patient populations and in long-term studies. The effect of acarbose on the incidence of type 2 diabetes is comparable to that of metformin, and in addition, acarbose is the only OAD that has been shown to reduce the risk of prespecified cardiovascular events irrespective of individuals' age and weight. Acarbose has been shown to improve hypertension and a number of cardiovascular risk factors, and may have long-term benefits for patients with metabolic syndrome. These properties can be attributed to the mode of action of acarbose, which directly targets postprandial hyperglycaemia and avoids several common side-effects associated with other antidiabetes medications. The most common side-effects that affect compliance with acarbose therapy are mild-to-moderate GI events, and these can be minimised if appropriate stepwise-dosing regimens are used at the start of treatment. However, acarbose has an excellent safety profile. Acarbose is therefore a valuable option for the management of type 2 diabetes and, as the only oral antidiabetes agent approved for the treatment of prediabetes, can help to improve clinical management across the dysglycaemic disease continuum.

## Conflict of interests

Professor Hanefeld has received speaking honoraria from Bayer Healthcare AG.
